# Human Placental Syncytiotrophoblasts Restrict *Toxoplasma gondii* Attachment and Replication and Respond to Infection by Producing Immunomodulatory Chemokines

**DOI:** 10.1128/mBio.01678-17

**Published:** 2018-01-09

**Authors:** Stephanie E. Ander, Elizabeth N. Rudzki, Nitin Arora, Yoel Sadovsky, Carolyn B. Coyne, Jon P. Boyle

**Affiliations:** a Department of Pediatrics, University of Pittsburgh School of Medicine, Pittsburgh, Pennsylvania, USA; b Center for Microbial Pathogenesis, Children’s Hospital of Pittsburgh of UPMC, Pittsburgh, Pennsylvania, USA; c Department of Biological Sciences, Dietrich School of Arts and Sciences, University of Pittsburgh, Pittsburgh, Pennsylvania, USA; d Magee-Women’s Research Institute, University of Pittsburgh School of Medicine, Pittsburgh, Pennsylvania, USA; e Department of Obstetrics, Gynecology, and Reproductive Science, University of Pittsburgh School of Medicine, Pittsburgh, Pennsylvania, USA; Stanford University

**Keywords:** CCL22, syncytiotrophoblast, *Toxoplasma gondii*, placenta

## Abstract

*Toxoplasma gondii* is a major source of congenital disease worldwide, but the cellular and molecular factors associated with its vertical transmission are largely unknown. In humans, the placenta forms the key interface between the maternal and fetal compartments and forms the primary barrier that restricts the hematogenous spread of microorganisms. Here, we utilized primary human trophoblast (PHT) cells isolated from full-term placentas and human midgestation chorionic villous explants to determine the mechanisms by which human trophoblasts restrict and respond to *T. gondii* infection. We show that placental syncytiotrophoblasts, multinucleated cells that are in direct contact with maternal blood, restrict *T. gondii* infection at two distinct stages of the parasite lytic cycle—at the time of attachment and also during intracellular replication. Utilizing comparative transcriptome sequencing (RNA-seq) transcriptional profiling, we also show that human placental trophoblasts from both the second and third trimesters respond uniquely to *T. gondii* infection compared to trophoblast cell lines, typified by the upregulation of several immunity-related genes. One of the most differentially induced genes was the chemokine CCL22, which relies on the secretion of a parasite effector(s) either during or after invasion for its induction. Collectively, our findings provide new insights into the mechanisms by which the human placenta restricts the vertical transmission of *T. gondii* at early and late stages of human pregnancy and demonstrate the existence of at least two interferon-independent pathways that restrict *T. gondii* access to the fetal compartment.

## IMPORTANCE

*Toxoplasma gondii* is a major source of congenital disease worldwide and must breach the placental barrier to be transmitted from maternal blood to the developing fetus. The events associated with the vertical transmission of *T. gondii* are largely unknown. Here, we show that primary human syncytiotrophoblasts, the fetus-derived cells that comprise the primary placental barrier, restrict *T. gondii* infection at two distinct stages of the parasite life cycle and respond to infection by inducing a unique immunomodulatory transcriptional profile. Collectively, our findings provide important insights into the mechanisms by which human syncytiotrophoblasts restrict *T. gondii* infection at early and late stages of human pregnancy, identify both permissive and resistant human placental cell types, and identify the placenta-enriched signaling pathways induced in response to infection.

## INTRODUCTION

*Toxoplasma gondii* is a major source of congenital disease, with ~200,000 global cases of congenital toxoplasmosis reported each year (1). In the majority of instances (~80%), *in utero* infections by *T. gondii* result in a range of severe birth defects, including ocular disease and developmental delays, and can also result in fetal death (2). However, despite the clear impact of *T. gondii* infections on fetal health, the mechanisms by which the parasite is transmitted from the maternal bloodstream into the fetal compartment are largely unknown.

In eutherian organisms, the placenta serves as the sole source of gas, nutrient, and waste exchange between the maternal and fetal compartments and acts as a key barrier to restrict fetal infections. At the forefront of these defenses is the syncytiotrophoblast (SYN), a multinucleated cell layer that comprises the outermost layer of the human placenta and which is in direct contact with maternal blood. Subjacent to the SYN layer are cytotrophoblasts (CYTs), mononucleated and proliferative cells that fuse to replenish the SYN layer throughout pregnancy. Together, these trophoblast layers form a primary barrier to the passage of pathogens that may infect the fetus by the hematogenous route.

In general, the pathways that exist in the human placenta to limit the vertical transmission of microbes are poorly defined. Our previous studies in primary human trophoblast (PHT) cells have identified at least two potent antiviral pathways that restrict viral replication in trophoblasts (3, 4). However, these pathways do not appear to be relevant during infection with nonviral pathogens, including *T. gondii* (5). While studies in placental explants suggest that the SYN layer is not permissive to *T. gondii* infection (6), the mechanistic basis for SYN resistance is incompletely understood, as is whether the SYN layer mounts any innate defense in response to parasite exposure. Moreover, while placental explant models are useful in their recapitulation of placental structure, they are limited in their capacity to dissect trophoblast cell-type-specific pathways that might exist to limit *T. gondii* infection.

In this study, we interrogated the trophoblast cell type specificity of *T. gondii* infection utilizing PHT cells isolated from full-term placentas and identified two cellular mechanisms that mediate SYN-specific resistance to *T. gondii* infection. In addition to discovering that SYNs restrict *T. gondii* attachment and replication while CYTs do not, we also identified cell signaling pathways uniquely induced by parasite infection in PHT cells, which included the induction of several immunity-related genes. We show that the majority of transcriptional changes in PHT cells are specific to the human pathogen *T. gondii* and do not occur in response to infection with the closely related parasite *Neospora caninum* (which is not known to cause disease in humans). Moreover, we show that the production of one such uniquely induced immunity-related gene, the T regulatory (T-reg) chemoattractant CCL22, is dependent on host cell invasion and the secretion of *T. gondii* effectors into the host cell. To expand our findings to earlier in human pregnancy, when the fetus is likely to face the more severe consequences of congenital *T. gondii* infections, we also utilized a midgestation chorionic villous explant model and show that second-trimester SYNs also resist *T. gondii* attachment and induce CCL22 in response to infection, whereas the fetus-derived amnion and chorion are permissive to infection and do not induce CCL22. Taken together, we have identified previously unknown intrinsic features in primary human placental cells from both the second and third trimesters of pregnancy that limit *T. gondii* infectivity at the level of attachment and replication, and our findings provide details on both host- and parasite-specific transcriptional responses of placental cells to infection.

## RESULTS

### Syncytiotrophoblasts isolated from full-term placentas resist *T. gondii* infection.

We found that PHT cells isolated from full-term placentas exhibited reduced susceptibility to *T. gondii* infection compared to primary human foreskin fibroblast (HFF) cells (see Fig. S1A and B in the supplemental material). These data are consistent with our previous work demonstrating that PHT cells exhibit reduced susceptibility to infection by the three major types of *T. gondii* in North America and Europe compared to nonplacental cells (7). Importantly, human trophoblast cell lines (including BeWo, HTR8, and JEG-3 cells) were unable to recapitulate this restrictive phenotype and were permissive to parasite infection (Fig. S1C). In addition, this phenotype was specific to PHT cultures as primary placental fibroblasts were as permissive to infection as were HFF cells (Fig. S1D).

10.1128/mBio.01678-17.1 FIG S1  (A and B) Immunofluorescence microscopy of HFF and PHT cultures inoculated with *T. gondii* RH strain (green) at a multiplicity of infection of 2 for 24 h. (A) Representative images of HFF (left) and PHT (right) cultures. Actin is shown in red; DAPI is shown in blue. (B) Ratio of parasite to host cell areas based on immunofluorescence of five fields of view per culture; single PHT preparation. *P* = 0.0064 based on two-tailed *t* test. (C and D) Growth curves of *T. gondii* CEP strain at a multiplicity of infection of 0.5 in the indicated cell types, as measured by luciferase expression by parasites. Growth over time is indicated in relative light units (RLU) as normalized to expression at 4 h postinfection and represented by the mean for three samples plus standard deviation. (C) *T. gondii* growth in three different trophoblast cell lines (BeWo, HTR8, and JEG-3) compared to HFF cells. (D) Comparison of *T. gondii* growth in primary cultures of HFF and PF (placental fibroblasts). (E) *T. gondii* (CEP) growth in HFF and BeWo cultures with or without 10 μM forskolin pretreatment at a multiplicity of infection of 0.5 as measured by luciferase expression by parasites. Growth over time is indicated in relative light units (RLU) as normalized to expression at 4 h postinfection and represented by the mean for three samples plus standard deviation. At least two biological replicates were performed. Download FIG S1, TIF file, 26 MB. Copyright © 2018 Ander et al. 2018 Ander et al. This content is distributed under the terms of the Creative Commons Attribution 4.0 International license.

**FIG 1:**
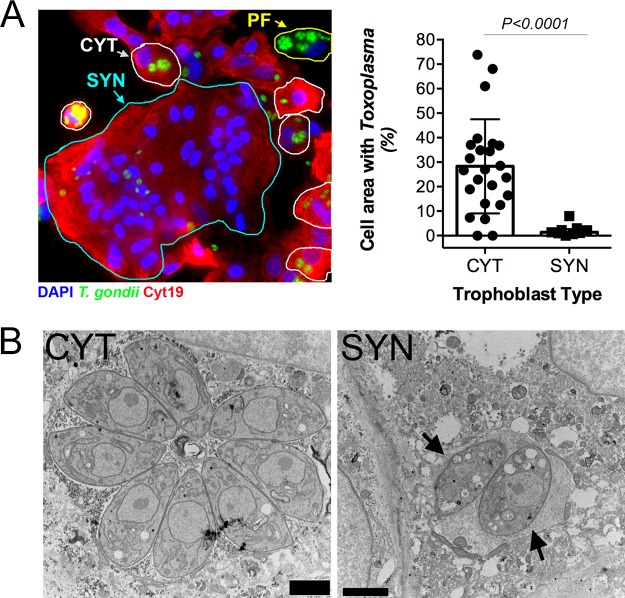
Placental syncytiotrophoblasts resist *T. gondii* infection. (A) (Left) Immunofluorescence microscopy of PHT cells inoculated with *T. gondii* RH strain (green) for ~24 h. Cytokeratin 19 is shown in red, DAPI is shown in blue, SYN is outlined in cyan, CYT is outlined in white, and the placental fibroblast (PF) is outlined in yellow. PF cells are distinguished by the lack of cytokeratin 19 (red). (Right) Percentage of cell area occupied by *T. gondii*, compared between CYT and SYN. *n*_CYT_ = 24 and *n*_SYN_ = 9, from one preparation of PHT cells. Two-tailed *t* test, *P* < 0.0001, with Welch correction for unequal variances. (B) Transmission electron microscopy of PHT cells infected with *T. gondii* (RH) for ~23 h. Mononucleated CYT at left and SYN, identified by its more than two nuclei, at right. Arrows indicate the parasites in the SYN. Bars, 2 μm.

PHT cells isolated from full-term placentas spontaneously fuse to form SYNs during their culture period (~72 h), with some retaining a mononuclear CYT phenotype. Therefore, to determine whether the lack of PHT cell infection occurred in a cell-type-specific manner, we infected PHT cells with yellow fluorescent protein (YFP)-tagged *T. gondii* (RH strain) and quantified parasite growth specifically in CYTs versus SYNs. These studies revealed dramatic differences in the susceptibilities of SYNs and CYTs to *T. gondii* infection—whereas CYTs were permissive to infection, SYNs were highly resistant (Fig. 1A, left). Furthermore, we observed that parasites within SYNs replicated to a lesser degree, as indicated by a highly significant reduction in total cell area occupied by parasites (Fig. 1A). Since *T. gondii* replicates within a parasitophorous vacuole (PV) generated at the time of invasion by each individual parasite, the number of parasites within a PV can serve as an indicator of parasite growth and replication. In contrast to the PVs in CYTs, those in SYNs most often contained 1 to 2 parasites (Fig. 1A, right). Importantly, fusion of BeWo cells with forskolin, which induces syncytin-mediated fusion (7), was not sufficient to confer resistance to *T. gondii* infection (Fig. S1E), supporting the idea that this phenomenon is specific to primary cells and does not rely on fusion alone.

Transmission electron microscopy (TEM) revealed that, whereas parasite growth and PV morphology were normal in mononucleated cells within the preparation (which are likely CYTs but could also be rare contaminating placental fibroblasts), SYN-internalized parasites were found within PVs containing host cell cytoplasmic contents indicative of a loss of vacuole integrity (Fig. 1B). Moreover, the parasites within these PVs contained more vacuoles of minimal electron density and poorly defined organelles (Fig. 1B). This phenotype is reminiscent of drug-induced death that we observed previously after treatment with a benzodioxole-containing compound (8), indicating that SYNs have potent toxoplasmacidal activity. Taken together, these data implicate SYNs as an innately resistant cellular barrier to *T. gondii* infection and suggest that unlike other cultured cells, these cells potently resist *T. gondii* infection.

### SYN-mediated resistance to *T. gondii* infection occurs at two stages of the parasite lytic cycle.

**FIG 2:**
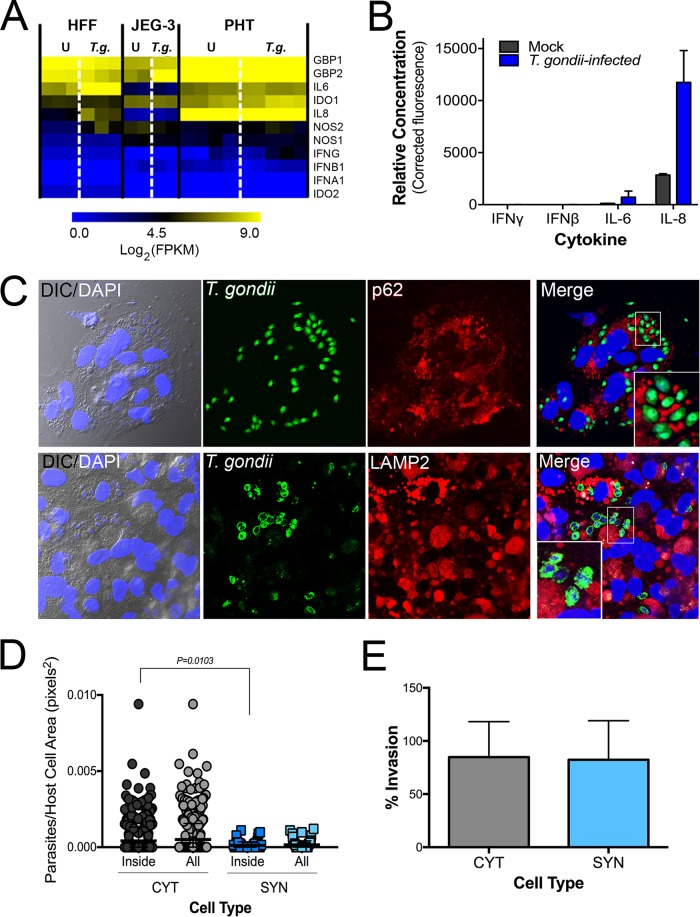
SYN-mediated restriction of *T. gondii* infection is not the result of autophagy, lysosomal degradation, or inability to invade. (A) Heat map of innate immune effector gene expression as determined by RNA-seq of uninfected (U) and *T. gondii* (*T.g.*)*-*infected HFF, JEG-3, and PHT cells. FPKM, fragments per kilobase per million. (B) PHT cytokine secretion as detected by Luminex assay of media from mock- and *T. gondii*-infected PHT cells. Data shown are from one of two PHT preparations. (C) Immunofluorescence microscopy of PHT cells infected with *T. gondii* (RH) (green) at 8 h postinfection. (Top) Infection with YFP-RH at a multiplicity of infection of 10; p62 staining is shown in red. (Bottom) Infection with RH (anti-GRA2, green) at a multiplicity of infection of 2; lysosome-associated membrane protein 2 (LAMP2) is shown in red; DAPI is shown in blue. (D and E) Quantification of inside/outside staining of *T. gondii* (YFP-RH)-infected PHT cells at 2 h postinfection at a multiplicity of infection of 1 in one PHT cell preparation. Using DIC/DAPI images, cells were classified as mononucleated (CYT) or multinucleated (SYN). (D) To compare the attachment efficiencies between cell types, the number of internalized parasites per two-dimensional cell area was calculated for 813 cells (*n*_CYT_ = 729, *n*_SYN_ = 84) and the resulting distributions were compared using the Kolmogorov-Smirnoff test. The comparison used was CYT_inside_ versus SYN_inside_ (*P* = 0.010). (E) Comparison of percent invaded parasites of all parasite-associated cells by cell type (*n*_CYT_ = 176, *n*_SYN_ = 34).

Our data suggest that SYNs restrict *T. gondii* infection at a stage of intracellular parasite growth. The primary mechanisms for cell-autonomous immunity to *T. gondii* are driven by the effector cytokine gamma interferon (IFN-γ). However, we found that uninfected and *T. gondii*-infected PHT cells had low levels of IFN-γ transcript (Fig. 2A) and that culture supernatants were devoid of secreted IFN-γ protein (Fig. 2A and B). Importantly, while guanylate binding protein 1 (GBP1) and GBP2 as well as other innate immunity-related factors (e.g., NOS1, NOS2, and indoleamine 2,3-dioxygenase [IDO]) have comparatively higher transcript levels in PHT cells (Fig. 2A), none of these well-characterized IFN-γ-driven host effector proteins were uniquely expressed in PHT cells (Fig. 2A). These findings suggest that the innate resistance of SYNs is not dependent on basal expression of IFN-γ or its stimulation by infection. To explore other related mechanisms, we performed immunofluorescence microscopy for markers of autophagy- and lysosome-mediated degradation pathways given the high level of basal autophagy that we previously observed in PHT cells (4). We found that there was no association between SQSTM/p62 or the lysosome-associated component LAMP2 and internalized parasites at either early or later stages of infection (Fig. 2C; Fig. S2). These findings are consistent with our TEM-based microscopic studies, in which we did not observe the association between double-membraned autophagosomes or lysosomes and internalized parasites (Fig. 1B).

10.1128/mBio.01678-17.2 FIG S2  Immunofluorescence microscopy of PHT cells infected with *T. gondii* (YFP-RH, multiplicity of infection of 4) (green). (A) LC3B staining is shown in yellow, actin is in red, and DAPI-stained nuclei are shown in blue at 8 h postinfection. (B) Lysosome-associated membrane protein 2 (LAMP2) is shown in red and DAPI is shown in blue at 24 h postinfection. Download FIG S2, TIF file, 7.7 MB. Copyright © 2018 Ander et al. 2018 Ander et al. This content is distributed under the terms of the Creative Commons Attribution 4.0 International license.

In addition to the intracellular control of parasite replication, it is possible that SYNs are protected from infection by defects in parasite attachment and/or invasion. To quantify parasite attachment and invasion, we performed a two-step immunofluorescence-based invasion assay to distinguish extracellular from intracellular parasites (as in reference 9 and other work). PHT cells were exposed to *T. gondii* (RH-YFP) for 2 h, at which point monolayers were washed to remove unbound parasites, cells were fixed, and attached parasites were identified using an antibody to surface antigen 1 (SAG1) in the absence of cell permeabilization, followed by detection using a secondary antibody conjugated to Alexa Fluor 594. Samples were then permeabilized and incubated again with anti-SAG1 antibody, which was detected with a secondary antibody conjugated to Alexa Fluor 633. Using this approach, extracellular parasites exhibited fluorescence in all channels (YFP, 594, and 633), whereas intracellular parasites exhibited fluorescence in only two (YFP and 633). Using differential interference contrast (DIC) imaging and automated image analysis, we quantified the extent of attached and internalized parasites in CYTs versus SYNs, which were easily distinguishable using DIC based upon the number, size, and clustering of their nuclei. Using this approach, we found that there were significantly fewer parasites overall (i.e., uninvaded and invaded) associated with SYNs than with CYTs (normalized for cell area; *P* = 0.010) (Fig. 2D). However, the percentages of invasion events (of all total parasite associations) were nearly identical between SYNs and CYTs, demonstrating that while there is a significant defect in parasite attachment to and/or association with SYNs, there is no obvious impediment to invasion (Fig. 2E). While we do not know the stage of attachment at which *T. gondii* tachyzoites are arrested when associating with SYNs compared to CYTs, these data point to a defect in *T. gondii* attachment as a primary mediator of SYN resistance to infection in addition to their ability to potently resist parasite replication when the parasites do successfully invade.

### PHT cells have a unique response to *T. gondii* infection characterized by the induction of immunity-related transcription factors and chemokines.

**FIG 3:**
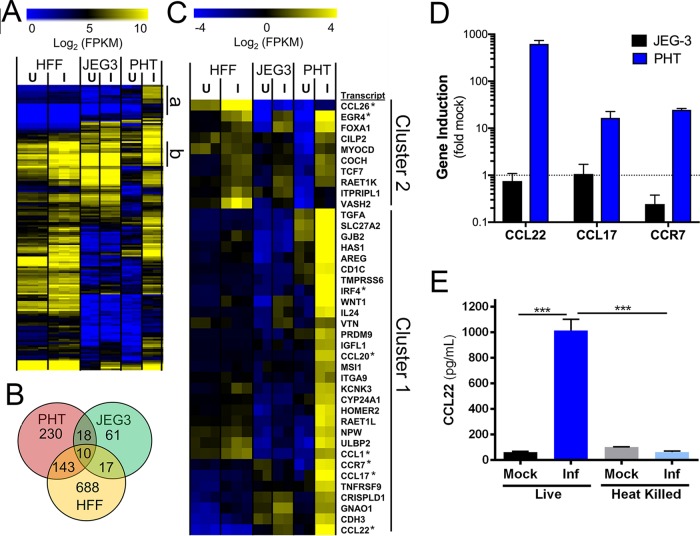
PHT cells infected with *Toxoplasma gondii* have a unique transcriptional response to infection. (A) Heat map of all genes with significantly higher transcript abundance in *T. gondii*-infected (I) PHT cells than in mock-infected (U) cells (*P* < 0.01; fold change, >4). (B) Genes with significantly higher abundance (*P* < 0.01; fold difference, ≥4) in PHT cells, HFFs, and JEG-3 cells. (C) Hierarchically clustered heat map of 40 genes induced in infected PHT cells. Cluster 1 contains genes that are induced in other cell types, while cluster 2 consists primarily of transcripts induced by *T. gondii* infection only in PHT cells (27/30). Transcription factors and chemokines and their receptors are indicated with asterisks. (D) qPCR validation of three genes specifically induced in *T. gondii*-infected PHT cells as identified by RNA-seq. Data shown consist of three technical replicates from an independent PHT cell preparation. (E) Induction of CCL22 secretion in PHT cells requires live parasites. Parasites were incubated at 23°C or 65°C for 1 h prior to being used to infect PHT cells. Data shown consist of two technical replicates from one PHT cell preparation. ***, *P* < 0.001 following one-way analysis of variance and multiple-comparison *post hoc* tests.

Given the dramatic differences in infectivity and growth of *T. gondii* in PHT cells, we infected PHT cells or the choriocarcinoma JEG-3 cell line with *T. gondii* and compared their transcriptional responses to infection using transcriptome sequencing (RNA-seq). Following infection for 24 h, we identified 401 transcripts of significantly different abundance (*P* < 0.01; fold change, >4) in infected PHT cells and 106 transcripts of different abundance in infected JEG-3 cells (Fig. 3A and B). To identify which transcripts were uniquely induced in PHT cells compared to JEG-3 cells and another primary cell line, we compared these data to a recently published RNA-seq data set from *T. gondii*-infected primary HFF cells (10). While we identified 858 host cell transcripts that were of different abundances in *T. gondii*-infected HFFs, there was a significant lack of overlap between infection-altered transcripts in HFF and PHT cells (Fig. 3B). Cluster analysis of all genes induced in *T. gondii*-infected PHT cells revealed multiple categories of genes specifically induced in these cells and not in either HFFs or JEG-3 cells. While some genes were induced uniquely in PHT cells and were expressed poorly in other cell types (Fig. 3A, cluster a), others were of high abundance only after infection in PHT cells but were constitutively expressed in other cell lines/types (Fig. 3A, cluster b). Focusing on genes uniquely induced in PHT cells compared to other cell types (cluster 1, Fig. 3C), multiple immunity-related transcription factors (e.g., IRF4 and EGR4), chemokines (CCL22, CCL17, CCL20, and CCL1) and the chemokine receptor CCR7 were all significantly induced by *T. gondii* infection. We confirmed this cell-type-specific induction of a subset of the cluster 1 genes via reverse transcriptase quantitative PCR (RT-qPCR) (Fig. 3D). Of note, we found that CCL22, a chemokine known to be expressed constitutively during pregnancy (11, 12) and that has also been found to increase during miscarriage (11), was induced by >400-fold in infected PHT cells based on RNA-seq and confirmatory RT-qPCR (Fig. 3C and D). Subsequent experiments with heat-killed *T. gondii* failed to induce CCL22 secretion from PHT cells (Fig. 3E), indicating that production of this chemokine requires live parasites and suggesting that the CCL22 response requires parasite invasion. Additional experiments treating PHT cultures with either neutralizing antibody to CCL22 or recombinant CCL22 showed no difference in the infection status of SYNs (Fig. S3), providing support for the fact that CCL22 is not responsible for the reduced parasite attachment to or replication within SYNs compared to CYTs. This finding is consistent with the role of CCL22 as a chemoattractant rather than an effector cytokine like IFN-γ.

10.1128/mBio.01678-17.3 FIG S3  PHT cells were superinfected with 2.16 × 10^5^ YFP-RH parasites for 24 h and stained with cytokeratin 19, phalloidin, and DAPI in order to distinguish cell type and boundary. Degree of infection was determined by parasite area as percentage of host cell, from images taken of two technical replicates from one PHT preparation. (A) PHT cultures were treated with a neutralizing antibody to CCL22 at the time of infection. (B) Cultures were pretreated with 1 ng/ml of rCCL22 for 24 h prior to infection. Download FIG S3, TIF file, 3.5 MB. Copyright © 2018 Ander et al. 2018 Ander et al. This content is distributed under the terms of the Creative Commons Attribution 4.0 International license.

### Infection of PHT cells with *Neospora caninum* does not induce the production of immunomodulatory chemokines.

**FIG 4:**
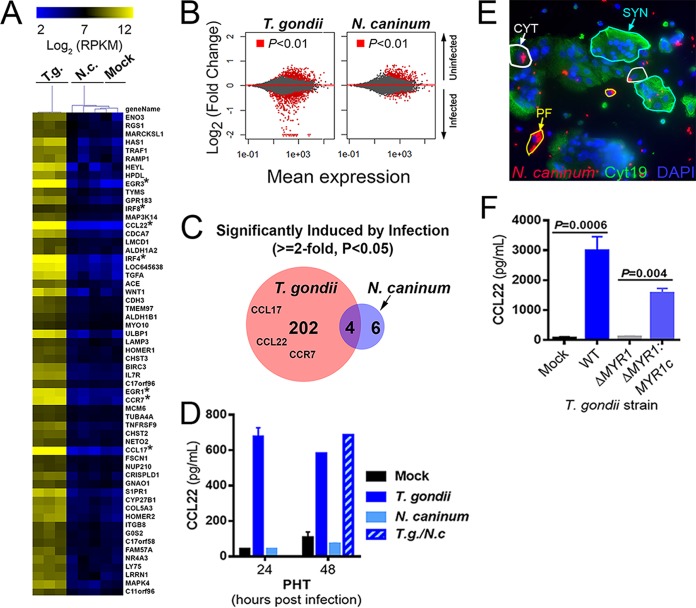
Infection-modulated gene expression is specific to *T. gondii* and requires parasite effectors. (A) Heat map of 59 genes induced by at least 1.8-fold in PHT cells (*P* < 0.01) after infection with *T. gondii* (T.g.) RH strain (multiplicity of infection of 3). Raw count data were converted to normalized RPKM using DESeq2. Data are also shown for *N. caninum* (N.c.)-infected (*n =* 3) and mock-infected (*n =* 2) PHTs, and mean centered data were clustered by sample and gene using the Euclidean distance (implemented in MeViewer [TM4 microarray suite]; see Materials and Methods). Asterisks denote genes that are very highly induced in *T. gondii*-infected PHT cells. (B) MA plots of PHTs comparing gene expression profiles in mock- and parasite-infected cells for all 23,735 queried genes. Genes of higher abundance in uninfected cells are indicated by positive changes, and those of higher abundance in infected cells are indicated by negative changes. (C) In *T. gondii*-infected PHTs, 206 genes were found to be of higher abundance than in mock-infected cells, while only 10 such genes were found in *N. caninum*-infected PHTs, consistent with the MA plots in panel B above. (D) Results from ELISA showing induction of CCL22 secretion in PHTs infected with *T. gondii* but not *N. caninum*. Host cells were infected with a multiplicity of infection of 2 (for each parasite species), and supernatants were harvested at the indicated time points. *n =* 2 to 3 wells for all treatments except for 48-h *T. gondii* and *T. gondii*/*N. caninum* (*n =* 1); single PHT cell preparation. (E) PHTs were infected with NC-1-dsRED *N. caninum* (multiplicity of infection of 3) for 24 h and stained with cytokeratin 19 antibodies and DAPI. Similarly to *T. gondii*, *N. caninum* grew efficiently in PFs (yellow outlines) and CYTs (white outlines) and poorly or not at all in SYNs (blue outlines). (F) CCL22 induction in PHT cells requires MYR1. PHTs were infected with either wild-type (WT) RH-YFP *T. gondii*, RHΔMYR1, or RHΔMYR1 complemented with a hemagglutinin-tagged copy of MYR1 (RHΔMYR1c). *n =* 3 for each *T. gondii* strain. Data shown are from one PHT cell preparation.

Host transcriptional responses to infection with *T. gondii* have been shown in a variety of cell types to be specific for *T. gondii* and are not associated with infection by one of its apicomplexan relatives, *Neospora caninum* (13–15). Unlike *T. gondii*, *N. caninum* is not a human pathogen but causes significant mortality in cattle and dogs and is associated with congenital disease in these animals (16, 17). To determine the specificity of the host response to *T. gondii* infection in PHT cells, we infected cells with *T. gondii* (RH-YFP) or *N. caninum* (NC-1-dsRED [18]) and compared the cellular responses to infection using RNA-seq. We found that *N. caninum* failed to significantly induce any of the chemokine/chemokine receptor genes that were induced by infection with *T. gondii* of PHT cells (Fig. 4A; asterisks indicate focus chemokine genes). The remarkable lack of differential transcript abundance in *N. caninum*-infected PHT cells compared to matched *T. gondii*-infected PHTs was further illustrated by MA plot (log ratio versus abundance) (Fig. 4B). In PHT cells, we identified 206 genes that were significantly induced by *T. gondii* infection (*P* < 0.05; fold induction, >2) and only 10 genes that were significantly induced after *N. caninum* infection (Fig. 4C). Consistent with this, infection with *N. caninum* had no effect on CCL22 levels, and coinfections with *N. caninum* and *T. gondii* showed that there was also no synergistic or additive effect (Fig. 4D). Importantly, despite the significant differences in gene induction, we found that PHT cells were similarly susceptible to *N. caninum* infection, with CYTs being readily invaded and supportive of parasite growth, while SYNs exhibited reduced invasion by and growth restriction of *N. caninum* in a fashion very similar to that observed for *T. gondii* (Fig. 4E).

### CCL22 induction in PHT cells requires the *T. gondii* dense granule protein MYR1.

Given that CCL22 induction in PHT cells required live parasites and was not induced by *N. caninum*, we reasoned that this induction likely resulted from a *T. gondii*-specific parasite effector that would be secreted after host cell invasion. *T. gondii* MYR1 is a recently identified dense granule protein that is required for the export/secretion of multiple dense granule effectors (including GRA24, GRA25, and GRA16 [14, 19, 20]) and was discovered based on its role in mediating *T. gondii*-specific activation of the transcription factor c-Myc (13). When we infected PHT cells with *T. gondii* lacking MYR1 (RH-Δ*MYR1*) and complemented control parasites (RH-Δ*MYR1*-*MYR1c*), we found that CCL22 production by PHT cells was entirely dependent upon MYR1 (Fig. 4F), which provides strong evidence that *T. gondii* CCL22 induction in PHT cells is driven by a MYR1-dependent secreted effector(s).

### Second-trimester human placental villi resist *T. gondii* infection and induce CCL22 in response to infection.

**FIG 5:**
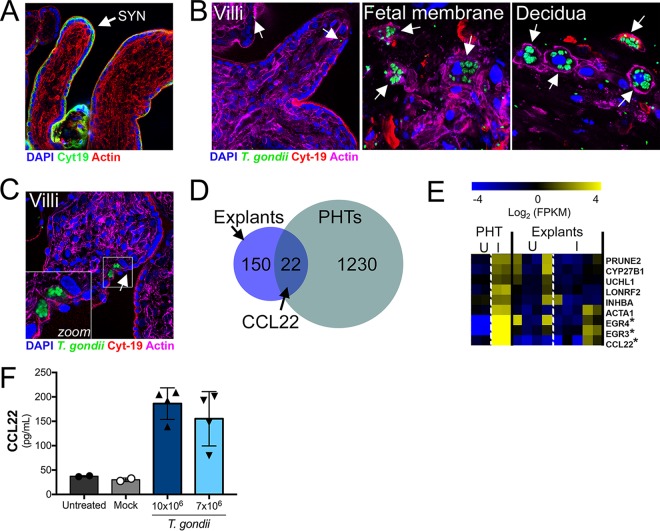
Chorionic villous explants from the second trimester are resistant to *T. gondii* infection and induce similar cytokine profiles in response to *T. gondii* as do full-term PHT cells. (A) Immunofluorescence microscopy of uninfected second-trimester placental villous explant with SYN localization indicated. Cytokeratin 19 in green; actin in red; DAPI in blue. (B) Immunofluorescence microscopy of midgestation villi (left panel), fetal membrane (middle panel), or decidua (right panel) inoculated with 10^7^ *T. gondii* cells (RH strain) for 24 h. *T. gondii* in green; cytokeratin 19 in red; actin in magenta; DAPI in blue. White arrows denote single parasites in isolated villi and PVs in fetal membrane and decidua. (C) Immunofluorescence microscopy of midgestation villi inoculated with 10^7^ *T. gondii* cells (RH strain) for 24 h. *T. gondii* in green; cytokeratin 19 in red; actin in magenta; DAPI in blue. Zoomed image from white box denotes PVs in CYTs located beneath a tear in the SYN (white arrow). (D and E) RNA-seq analysis of *T. gondii*-infected villous explants. Venn diagram (D) and heat map (E) showing a subset of genes that are similarly induced in one of the *T. gondii*-infected villous explants (explant 5) and PHTs. Genes were selected if they were at least 2-fold induced compared to mock infection and the value was significant using a DESeq2-determined adjusted *P* value of <0.05. Explant data in heat map are from 3 genetically distinct placenta preparations, while PHT data shown are the same as in Fig. 3. I, infected; U, uninfected (mock); FPKM, fragments per kilobase per million. Asterisks denote genes similarly induced in both explant 5 and PHT cells. (F) ELISA showing *T. gondii* (RH strain)-mediated enhancement of CCL22 secretion from villous explants at 24 h postinfection.

Because PHT cells are isolated from full-term placentas, we next determined whether SYNs from earlier in human pregnancy also resist *T. gondii* infection and similarly induce immunity-related genes. To do this, we utilized second-trimester chorionic villous explants, which retain the morphology of human placental villi, including a layer of cytokeratin 19-positive SYNs covering the villous surfaces (Fig. 5A). Consistent with our findings in PHT cells, and the work of others utilizing first-trimester explants (6), we found that second-trimester SYNs were resistant to *T. gondii* infection, even when infected with very high numbers of parasites (10^7^) (Fig. 5B, left panel). This resistance appears to be primarily at the level of parasite attachment, as we detected very few internalized parasites in placental villi and most parasites detected appeared to be extracellular (Fig. 5B, left panel, white arrows). Importantly, unlike placental villi, we found that fetal membrane (amnion and chorion) and maternal decidua supported *T. gondii* replication (Fig. 5B, middle and right panels, respectively), highlighting the specific resistance of placental villi. In addition, consistent with the work of others utilizing first-trimester explants (6), we found that CYTs subjacent to the SYN were permissive to *T. gondii* only when the SYN layer was breached (Fig. 5C). These data show that the SYN layer also forms a barrier to *T. gondii* vertical transmission in midgestation.

Next, we profiled the transcriptional changes induced by *T. gondii* infection of second-trimester chorionic villi using RNA-seq to determine whether they responded to parasite infection similarly to PHT cells from late gestation. We found that 172 transcripts were differentially expressed in response to *T. gondii* infection of villous explants, with 22 of these transcripts also being differentially expressed in response to infection of PHT cells, which included EGR3, EGR4, and CCL22 (Fig. 5D and E). We confirmed that CCL22 was induced at the protein level by enzyme-linked immunosorbent assay (ELISA) in supernatants from *T. gondii*-infected second-trimester villi (Fig. 5F). These data suggest that CCL22 is specifically induced by the human placenta in response to *T. gondii* infection at both early and late stages of pregnancy.

## DISCUSSION

*T. gondii* infections present a worldwide threat to pregnant women, and there is an urgent need to develop novel treatment regimens to block congenital transmission of *T. gondii* and other pathogens that pose risks to the developing fetus. Our data presented here point to a direct role for SYN-intrinsic pathways in the protection of the fetus from *T. gondii* infection, making the SYN a highly unique cell type given that a diverse array of cells studied to date are susceptible to *T. gondii* infection and replication. A particularly remarkable feature of the PHT cell culture model is that there are both permissive and resistant cells in very close proximity, indicating that cell-autonomous features determine the fate of *T. gondii* upon interaction with CYTs and SYNs. Our studies in both human primary full-term and second-trimester SYNs suggest that these cells evade *T. gondii* infection at two critical stages of the parasite lytic cycle—at the point of parasite association with the host cell and during intracellular growth. Moreover, by comparing cell-type-specific transcriptional profiles from *T. gondii*-infected primary trophoblasts and placental tissue with those of other cell types, we identified unique sets of genes induced by *T. gondii* infection in the human placenta, including the induction of CCL22 that requires the presence of parasite-encoded MYR1. Collectively, our data provide significant advances in our understanding of how the human placenta controls, responds to, and is manipulated by *T. gondii* infection.

We found that the first point of SYN-mediated restriction of *T. gondii* infection occurred at the level of parasite association and/or attachment, which we observed in SYNs isolated both from full-term placentas and from midgestation chorionic villi. These findings are consistent with the previous work of others suggesting that attachment might be reduced in first-trimester SYNs (6), although this was not directly tested. Our attachment and invasion data from PHT cells provide direct evidence that SYNs naturally restrict parasite attachment but are susceptible to invasion once parasite attachment occurs, which appears to be a rare event. It remains unclear which point of the attachment process is altered when *T. gondii* associates with SYNs, but a likely stage is during the early phase of gliding motility prior to the second phase of attachment (which is mediated by the secretion of microneme and rhoptry organelles [21]). One possibility is that when parasites encounter SYNs, they glide less efficiently than on CYTs or placental fibroblasts, which ultimately results in significantly reduced “full” attachment (mediated by microneme and rhoptry secretions). Differences in membrane biochemistry in SYNs versus CYTs and fibroblasts could underlie these important differences in early parasite association. Differences in glycosaminoglycan (GAG) content between CYTs and SYNs could also explain these differences given the known importance of GAGs in parasite gliding motility and attachment (22). However, once secondary attachment occurs, invasion seems to proceed normally, indicating that whatever membrane/surface differences there are between CYTs and SYNs, they do not impact the phase of invasion that requires organelle discharge.

In midgestation chorionic villi, the poor association/attachment phenotype was even more profound than that observed in PHT cells, with little to no parasite association with the villi observed. These findings suggest that in addition to biochemical surface differences between SYNs and CYTs, the morphology of the SYN layer itself may directly impact parasite association and attachment. This could be influenced by morphological differences in this model, including the positive membrane curvature associated with the significant branching of the placental villous trees, which might impact lipid, protein, and/or carbohydrate composition. In addition, the apical surfaces of SYNs associated with placental explants may be more differentiated than PHT cells, which might impact parasite attachment through the presence of a highly dense brush border in the explant model. Consistent with this, we previously observed very little *T. gondii* association in a bead-based three-dimensional cell line model of human SYNs, which also induces significant membrane curvature and allows for the formation of a well-differentiated brush border (7).

For parasites that attach to the SYN layer, our data suggest a second level of resistance to infection that occurs postinvasion. Importantly, in contrast to other cell types, our data show that this resistance is not mediated by IFN-γ, which is not basally expressed in PHT cells or induced by *T. gondii* infection. Furthermore, we did not find any evidence for autophagy- or lysosome-mediated degradation pathways in the intracellular restriction of parasite replication. To date, all known “cell-autonomous” mechanisms of parasite killing in human cells rely on previous stimulation with IFN-γ. For example, IFN-γ can induce a variety of downstream effector mechanisms depending on the cell type, including tryptophan starvation in HFFs (23) and decoration of the vacuole with guanylate binding proteins (24, 25), ubiquitin, or other markers for autophagy, including LC3B (25, 26). Ultimately, these pathways would lead to lysosomal fusion with the parasite-containing vacuole and parasite destruction, which we did not detect in infected SYNs at any time point tested. However, our data conclusively show that parasites that invade this specific trophoblast cell type are able to form functional vacuoles but are ultimately destroyed in a fashion reminiscent of some toxoplasmacidal drugs (8, 27). Hallmarks of parasite killing are vacuolation of the parasites, breakdown of the vacuolar tubulovesicular network, and lack of integrity of the parasitophorous vacuolar membrane and leakage of host cytoplasmic contents into the lumen of the compromised vacuole. These data place PHT cells, and specifically the subpopulation of fused SYNs, into a rare class of cells that not only restrict the growth of *T. gondii* after invasion in the absence of any external stimuli but actively destroy invaded parasites. While we did not compare them head to head with SYNs, neutrophils restrict *T. gondii* growth after invasion but are possibly less toxoplasmacidal than SYNs, given that neutrophils have been implicated in the spread of *T. gondii* throughout the intestine in a murine model (28). Head-to-head comparisons between SYNs and innate immune cell types like neutrophils will help to illuminate what potential killing mechanisms might be shared, or not, between these cell types. Moreover, determining the differences between SYNs and CYTs would require the development of new methods to separate them for downstream analyses, including RNA-seq and quantitative proteomics.

In addition to resisting *T. gondii* infection, our data show that PHT cells robustly induce the chemokine CCL22 in response to infection by a MYR1-dependent effector secretion mechanism. We do not know which cell types within the PHT preparation produce CCL22 after exposure to *T. gondii*, but given the dependence upon successful invasion for this response, a good candidate cell type is the CYT rather than the SYN, although cell-specific analyses are required to address this directly. Importantly, the major inflammatory responses induced in PHT cells by *T. gondii* infection are not induced (or are induced much more poorly) after infection with *N. caninum*, a near relative of *T. gondii* that does not successfully infect humans or rodents, suggesting that it may be a host and/or parasite adaptation that may impact disease outcome.

The precise role of CCL22 in human pregnancy is unknown, but maternal cells express CCL22 at low levels throughout pregnancy, with increased levels associated with miscarriage (11). Moreover, the induction of chemokines, including CCL22 and CCL17, is associated with preterm birth in humans (29) and in small-animal models (30). The precise role played by CCL22 during *T. gondii* vertical transmission remains to be determined. However, exposure of PHT cells to recombinant CCL22 or CCL22-neutralizing antibody had no impact on parasite replication (see Fig. S3 in the supplemental material), supporting the idea that it has no direct antiparasitic activity. A likely scenario is that the induction of CCL22, and other immunity-related genes, is aimed at alerting the maternal immune system to placental infection, where it could play any number of roles in mediating the dialogue between maternal and fetal tissues, such as enhancing immune cell-mediated protection at the maternal-fetal interface or in terminating the pregnancy should levels reach a specific threshold. Given that CCL22 levels are elevated in maternal serum during healthy, infection-free pregnancies (11, 12), CCL22 and its recruitment of regulatory T cells may also play a role in immune tolerance throughout gestation, which is modulated in response to infection. This increased T-reg recruitment may even promote *T. gondii* infection, as was seen in *Listeria* and *Salmonella* infections in the pregnant mouse model (31).

Our findings provide important insights into the molecular and cellular pathways utilized by human SYNs at both late and middle stages of gestation to restrict *T. gondii* access to the fetal compartment. In addition, by characterizing the immunologic pathways induced by *T. gondii* infection of SYNs, our findings have uncovered potentially novel biomarkers of infection severity that might have important roles in shaping the maternal systemic immune response. These findings provide an example of the signaling cross talk that exists between the maternal and fetal compartments and the mechanisms by which this signaling is impacted by parasite-associated effectors. Taken together, these findings provide important insights into *T. gondii-*induced congenital disease that could lead to the design of novel therapeutics aimed at reducing congenital toxoplasmosis.

## MATERIALS AND METHODS

### Cell culture.

All cell and tissue cultures were incubated at 37°C and 5% CO_2_, and all media were supplemented with 10% fetal bovine serum (FBS) and 50 μg/ml penicillin-streptomycin. JAR and HTR8 cells were grown in RPMI 1640 medium (HyClone), BeWo cells were grown in F-12K medium (Corning), and JEG-3 cells were grown in Eagle’s minimum essential medium (EMEM; Lonza). To induce fusion of BeWo cells, cells were treated with 10 μM forskolin for 24 h and then washed with phosphate-buffered saline (PBS) before infection. Primary human trophoblast (PHT) cells were isolated from healthy, full-term pregnancies and were cultured as described previously (3, 4). PHT cells were cultured for ~48 h prior to infection to allow for SYN formation. Primary placental fibroblasts were isolated and cultured as described previously (32).

### Midgestation placental explants.

Human placental tissue from less than 24 weeks of gestation was obtained from the University of Pittsburgh Health Sciences Tissue Bank through an honest broker system after approval from the University of Pittsburgh Institutional Review Board and in accordance with the University of Pittsburgh anatomical tissue procurement guidelines. Chorionic villi, fetal membrane, and decidua were dissected and cultured in Dulbecco modified Eagle medium (DMEM)–F-12 medium (1:1) supplemented with 10% FBS, penicillin-streptomycin, and amphotericin B. For *T. gondii* infections, isolated tissue was infected immediately following isolation with 2.5 × 10^4^ to 1 × 10^7^ parasites for ~24 h. For imaging, tissue was fixed in 4% paraformaldehyde, and imaging was performed as detailed below.

### Parasites.

Type I (RH) and type III (CEP) *T. gondii* and *N. caninum* (NC-1) tachyzoites were used for this study. All parasites were maintained by continual passage in human foreskin fibroblast (HFF) cultures incubated at 37°C and 5% CO_2_ in DMEM supplemented with 10% FBS, 50 μg/ml penicillin-streptomycin, and 2 mM glutamine. The YFP-RH was a gift from David Roos, and the RH-*MYR1*-KO and RH-*MYR1*-KO/complemented parasites were gifted by John Boothroyd. For infections, infected monolayers were scraped and syringe lysed to release the tachyzoites. These parasites were then pelleted at 800 × *g* for 10 min, resuspended in fresh medium, filtered through a 5-μm filter, and counted to determine the appropriate dilution for infection. Mock inoculum was produced by filtering out the tachyzoites with an 0.2-μm filter.

Parasite growth curves were generated by luciferase assay (Promega) using luciferase-expressing CEP parasites. Briefly, at each time point, samples were lysed using the passive lysis buffer (Promega) and stored at −20°C until at least 8 h past the last time point collection. Samples were then thawed and incubated with substrate, and fluorescence was measured.

### RT-qPCR and RNA-seq.

RNA was isolated from cultures using the GenElute mammalian total RNA miniprep kit (Sigma) and the associated DNase digestion set (Sigma). Both a NanoDrop spectrophotometer and an Agilent bioanalyzer were used to determine sample quality. Sequencing libraries were prepared from 0.2 to 0.9 μg of total RNA with the TruSeq stranded mRNA library preparation kit (Illumina). The Illumina NextSeq 500 sequencer was used for sequencing. CLC Genomics Workbench 9 (Qiagen) was used to map the RNA-seq FASTQ reads to the human reference genome (hg19). Differential expression analysis was performed using the DESeq2 package in R (33) using a significance cutoff of an adjusted *P* value (*P*_adj_) of <0.01, unless specified otherwise. Analysis of mock- and *T. gondii*-infected HFF cells was based on data sets previously published and deposited in the Sequence Read Archive (SRA): SRR2644999, SRR2645000, SRR2645001, SRR2645002, SRR2645003, and SRR2645004. Untreated and mock-infected chorionic villous explant RNA-seq data sets were previously published and deposited in the SRA: SRR5676707, SRR5676716, SRR5676717, SRR5676703, and SRR5676704. Hierarchical clustering of log_2_-transformed read-per-kilobase-per-million (RPKM) data was performed using MeViewer TM4 software. Data were either clustered as is or linearly mean centered using Euclidian distance. Color scales were adjusted for presentation purposes.

For RT-qPCR analyses, RNA was isolated as described above and cDNA was generated using the iScript cDNA synthesis kit (Bio-Rad), followed by qPCR using a StepOnePlus real-time PCR system (Thermo Fisher). The threshold cycle (Δ*C*_*T*_) method was used to determine gene expression and normalized to the human actin *C*_*T*_ of each sample. Primer sequences were as follows: actin, ACTGGGACGACATGGAGAAAAA (forward, 5′ to 3′) and GCCACACGCAGCTC (reverse, 5′ to 3′), and CCL22, GTGGTGTTGCTAACCTTC (forward, 5′ to 3′) and GGCTCAGCTTATTGAGAATC (reverse, 5′ to 3′). Predesigned primers were ordered for CCL17 (Qiagen) and CCR7 (Sigma).

### Microscopy.

Cell monolayers and placenta explants were fixed in 4% paraformaldehyde and permeabilized with 0.1% Triton X-100 in 1× PBS. Primary antibodies were incubated for 1 h at room temperature, followed by washing, and then secondary antibodies were conjugated to Alexa Fluor (Invitrogen) fluorophores for 30 min at room temperature. Following washing, cells/explants were mounted with 4′,6-diamidino-2-phenylindole (DAPI)–Vectashield (Vector Laboratories), and imaging was performed on an Olympus FV1000 laser scanning confocal microscope, a Zeiss LSM 710 microscope, or an Olympus IX83 inverted microscope. In some cases, images were adjusted for brightness and contrast using Photoshop or Fiji/ImageJ. ImageJ was used for image analyses. Transmission electron microscopy was performed as described previously (4).

Reagents and antibodies used for immunostaining studies include Alexa Fluor 594- or 633-conjugated phalloidin (Invitrogen), cytokeratin 19 (Abcam), LAMP2 (Santa Cruz), and SAG1 (mouse monoclonal D61S; Thermo Fisher).

### CCL22 ELISA.

CCL22 ELISAs were performed with the human CCL22/MDC DuoSet ELISA (R&D Systems) per the manufacturer’s instructions.

### Luminex.

Conditioned medium from cells was analyzed by multiplex Luminex assay by the University of Pittsburgh Cancer Institute (UPCI) Cancer Biomarkers Facility Luminex Core Laboratory.

### Statistics.

All statistics were calculated using GraphPad Prism. Experiments were performed with independent preparations of PHT cells and second-trimester villous explants. The data are presented as the mean ± standard deviation (SD). The individual statistical analyses and associated *P* values are described in the individual figure legends.

### Accession number(s).

BioSample accession numbers: SAMN08172699, SAMN08172700, SAMN08172701, SAMN08172702, SAMN08172703, SAMN08172704, SAMN08172705, SAMN08172706, SAMN08172707, SAMN08172709, SAMN08172710, SAMN08172711, SAMN08172712, SAMN08172713, SAMN08172714, SAMN08172715, SAMN08177641, SAMN08177642, SAMN08177643, SAMN08177644.

BioProject accession number: PRJNA422277 (SRP126779).
