# Supplementary figures and images for: Human Placental Syncytiotrophoblasts Restrict Toxoplasma gondii Attachment and Replication and Respond to Infection by Producing Immunomodulatory Chemokines
Source: mBio. 2018 Jan 9;9(1):e01678-17. doi: 10.1128/mBio.01678-17 (PMC5760739; doi:10.1128/mBio.01678-17)

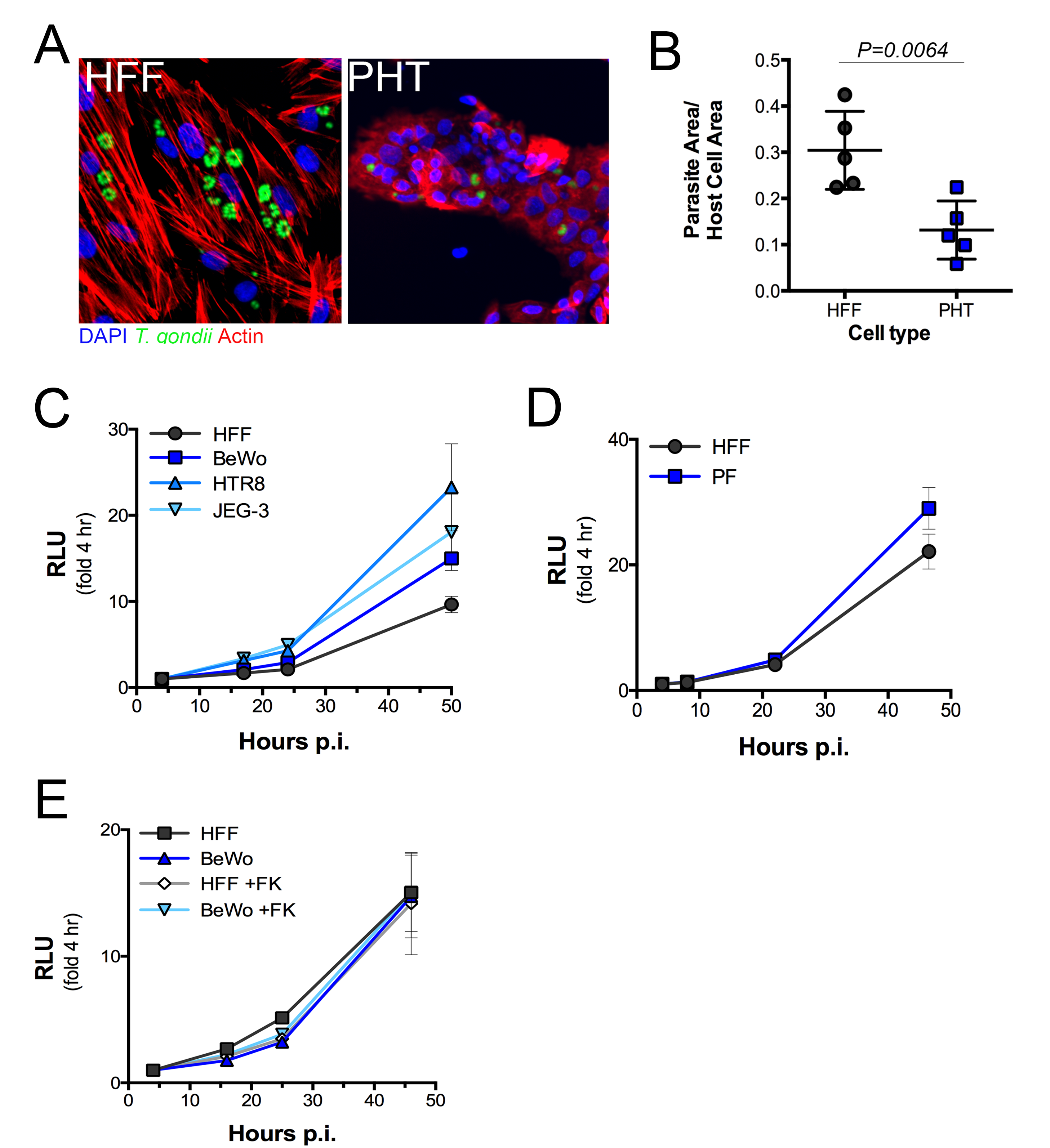

Supplement: FIG S1 [file mbo001183659sf1.tif]

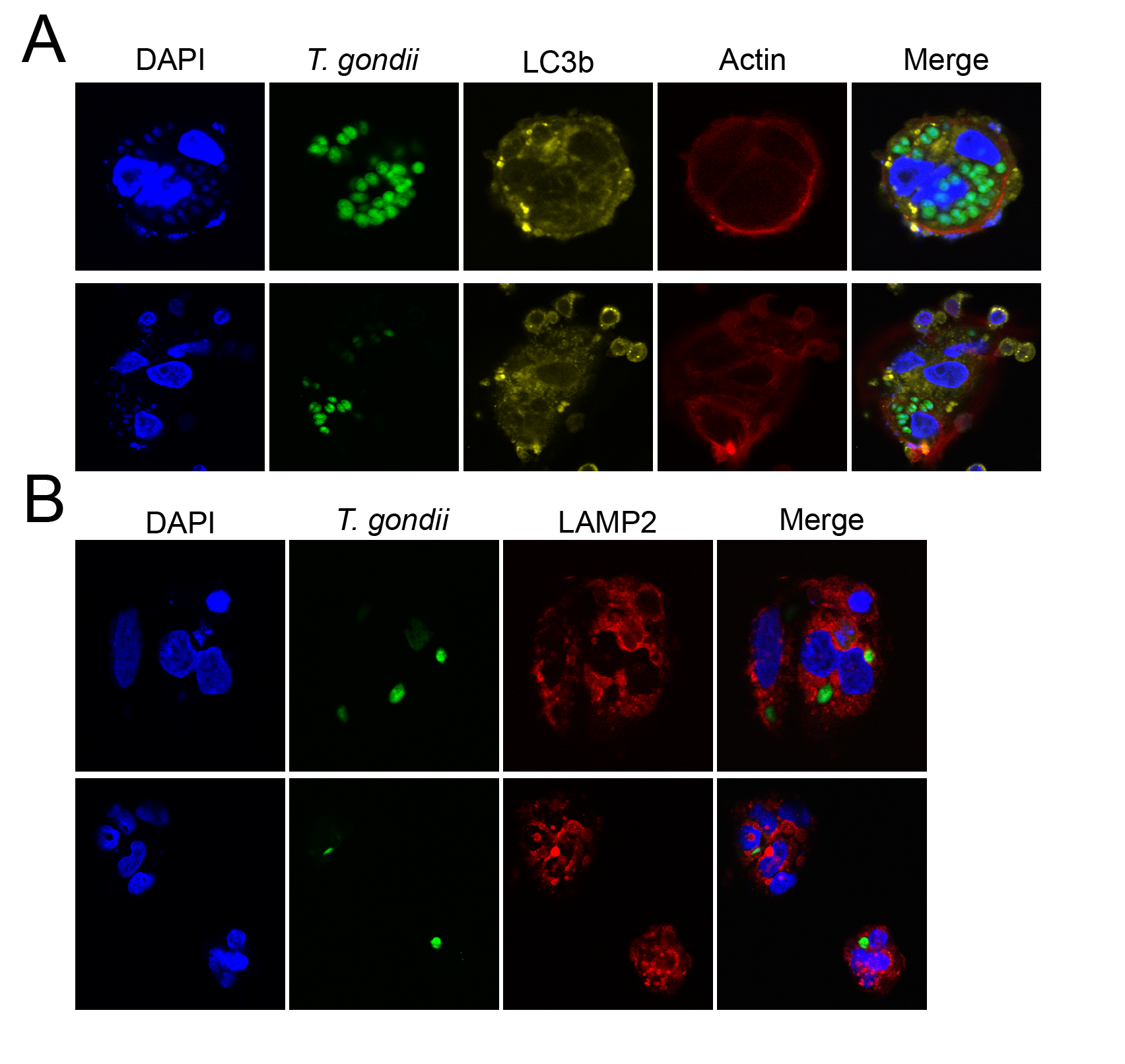

Supplement: FIG S2 [file mbo001183659sf2.tif]

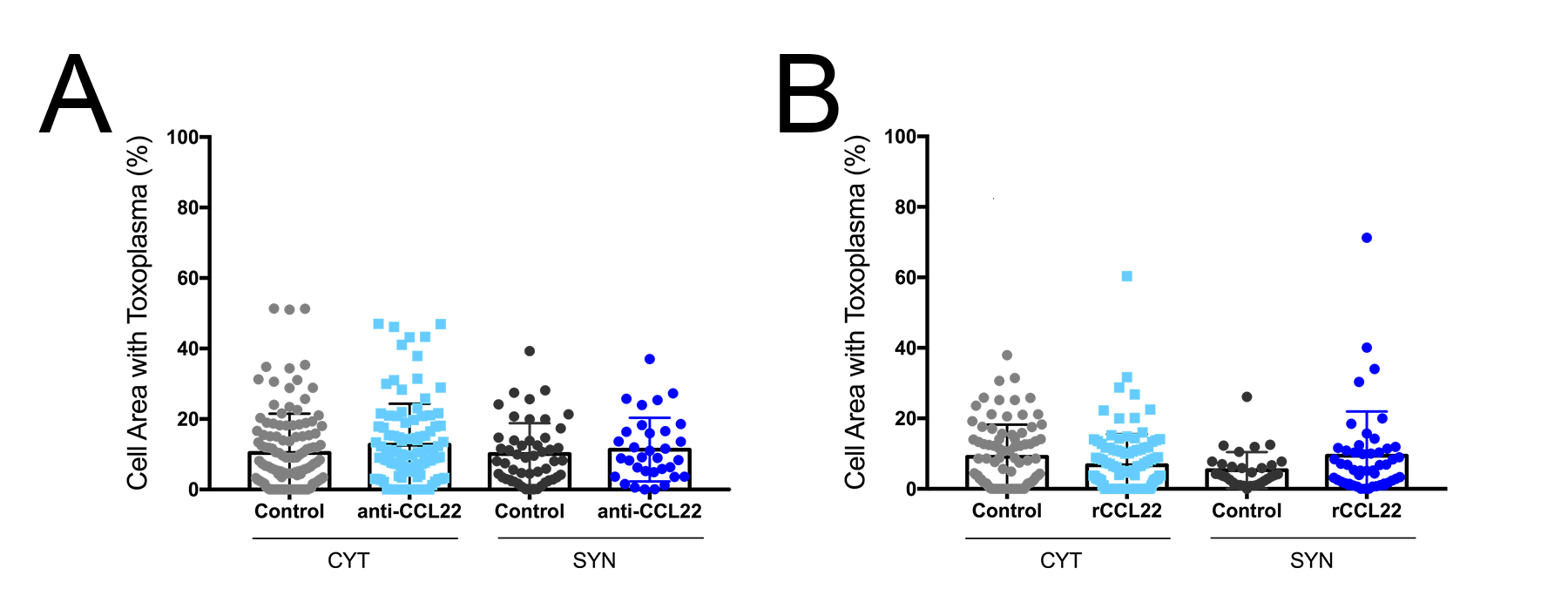

Supplement: FIG S3 [file mbo001183659sf3.tif]
